# Chemical Constituents of the Roots of *Polygala tenuifolia* and Their Anti-Inflammatory Effects

**DOI:** 10.3390/plants11233307

**Published:** 2022-11-30

**Authors:** So-Ri Son, Young-Seo Yoon, Joon-Pyo Hong, Jae-Min Kim, Kyung-Tae Lee, Dae Sik Jang

**Affiliations:** 1 Department of Biomedical and Pharmaceutical Sciences, Graduate School, Kyung Hee University, Seoul 02447, Republic of Korea; 2 Department of Pharmaceutical Biochemistry, College of Pharmacy, Kyung Hee University, Seoul 02447, Republic of Korea; 3 Department of Life and Nanopharmaceutical Sciences, Graduate School, Kyung Hee University, Seoul 02447, Republic of Korea; 4 Department of Pharmaceutical Science, College of Pharmacy, Kyung Hee University, Seoul 02447, Republic of Korea

**Keywords:** *Polygala tenuifolia*, oligosaccharide ester, inflammation, nitric oxide, prostaglandin E_2_, proinflammatory mediators

## Abstract

Increasing scientific evidence has demonstrated that the roots of *Polygala tenuifolia* Willd. have pharmacological effects related to anti-inflammation. Therefore, the aim of this study is to investigate the chemical constituents from *P*. *tenuifolia* roots as anti-inflammatory drug candidates. In the present work, twenty-three compounds were isolated from *P*. *tenuifolia* roots, including three saponins (**1**–**3**), ten phenylpropanoid sucrose esters (**4**–**12**), one benzoic acid sugar ester derivative (**13**), four xanthones (**14**–**17**), two hydroxy benzophenone derivatives (**18** and **19**), two phenolic derivatives (**20** and **21**), and two ionones (**22** and **23**). All isolates were tested for their inhibitory effects of LPS-stimulated NO and PGE_2_ production in RAW 264.7 macrophages. Among these, 3-*O*-(3,4,5-trimethoxy-cinnamoyl),6′-*O*-(*p*-methoxybenzoyl) sucrose ester (TCMB; **11**) together with compounds **3** and **21** exhibited significant inhibitory effects on NO production, while TCMB and compounds **17**, **19**, and **21** showed strong inhibitory effects on PGE_2_ production. Specifically, TCMB (**11**) downregulated the protein levels of iNOS and COX-2 in LPS-induced RAW 264.7 macrophages. In addition, TCMB (**11**) dose-dependently diminished the relative mRNA expression levels of iNOS, PGE_2_, and proinflammatory cytokines (TNF-α, IL-1β, and IL-6). A molecular docking study showed that TCMB (**11**) has strong binding affinities with iNOS and COX-2.

## 1. Introduction

Inflammation is one of the vital processes in which immune cells are activated by microbial infections, certain injuries, or toxins [1]. Macrophages play a crucial role in innate immune response by their cytotoxic and phagocytosis mechanism [2]. However, excessive immune response can lead to chronic inflammation, which in turn causes a variety of inflammatory-mediated disease [3]. Lipopolysaccharide (LPS), an endotoxin, is derived from Gram-negative bacteria and detected by Toll-like receptor 4 (TLR4) to activate the macrophages [4]. In activated macrophages by LPS, transcription factors of inflammatory response such as nuclear factor kappa-light-chain-enhancer of activated B cells (NF-κB) are consequently expressed. As a result, proinflammatory mediators such as nitric oxide (NO) and prostaglandin E_2_ (PGE_2_) are produced by inducible nitric oxide synthase (iNOS) and cyclooxygenase-2 (COX-2) [5]. NO is a signaling molecule in the immune process, but overproduction of NO can lead to inflammation diseases such as septic shock, cardiomyocyte apoptosis, and osteoarthritis [6,7,8]. PGE_2_ can also cause vasodilatation, edema, fever, and pain [9]. Therefore, controlling proinflammatory mediators is an important factor in the development of therapeutic agents for various inflammation-related diseases.

The genus *Polygala* is one of the largest genera in the family Polygalaceae, with more than 600 species occurring throughout the world [10]. Since ancient times, several species of the genus *Polygala* have been widely used as main ingredients in traditional herbal remedies. In particular, the roots of *Polygala tenuifolia* Willd (Polygalae Radix) are included in several standardized documents, including the European (Ph. Eur.), Korean, Chinese, and Japanese Pharmacopoeias, as well as WHO monographs. Polygalae Radix, also known as “Onji” or “Yuanzhi” in East Asia, is one of the most important ingredients in traditional Oriental medicine. For example, it has been extensively used to treat inflammation-mediated diseases, neurasthenia or anxiety-related disorders, and memory deficits [10]. Recent research has revealed that *P*. *tenuifolia* roots have pharmacological effects related to anti-inflammatory, antitumor, neuroprotective, antidepressant, and sedative–hypnotic activities [11,12,13,14,15]. In phytochemical studies, triterpenoids, oligosaccharide esters, and phenolic compounds, including xanthones, have been identified from *P. tenuifolia* roots [10]. In a project directed towards the search for anti-inflammatory compounds in plants, a 70% MeOH extract from the roots of *P. tenuifolia* showed a significant inhibitory effect on the production of LPS-induced NO in RAW 264.7 macrophages (inhibition rate of 32.66% at 100 μg/mL). Despite the fact that chemical constituents of *P*. *tenuifolia* have been identified as potent anti-inflammatory agents, only a few compounds have undergone significant research to date. Therefore, the aim of this study is to investigate chemical constituents of the roots of *P*. *tenuifolia* as anti-inflammatory drug candidates.

In the present work, secondary metabolites in the 70% MeOH extract of *P*. *tenuifolia* were isolated through repeated chromatographic purification. To investigate the anti-inflammatory properties of the isolates, they were incubated with LPS-stimulated RAW 264.7 macrophages. The most potent compound was chosen to identify its effects on iNOS/COX-2 proteins and mRNA expression. The inhibitory activity of this compound on proinflammatory mediator mRNAs, including TNF-α, IL-1β, and IL-6, was also tested. Finally, a molecular docking study with iNOS and COX-2 protein was conducted to assess the performance of the compound as a selective iNOS and COX-2 inhibitor.

## 2. Results

### 2.1. Isolation and Identification of Compounds ***1**–**23*** from the Roots of P. tenuifolia

**Figure 1:**
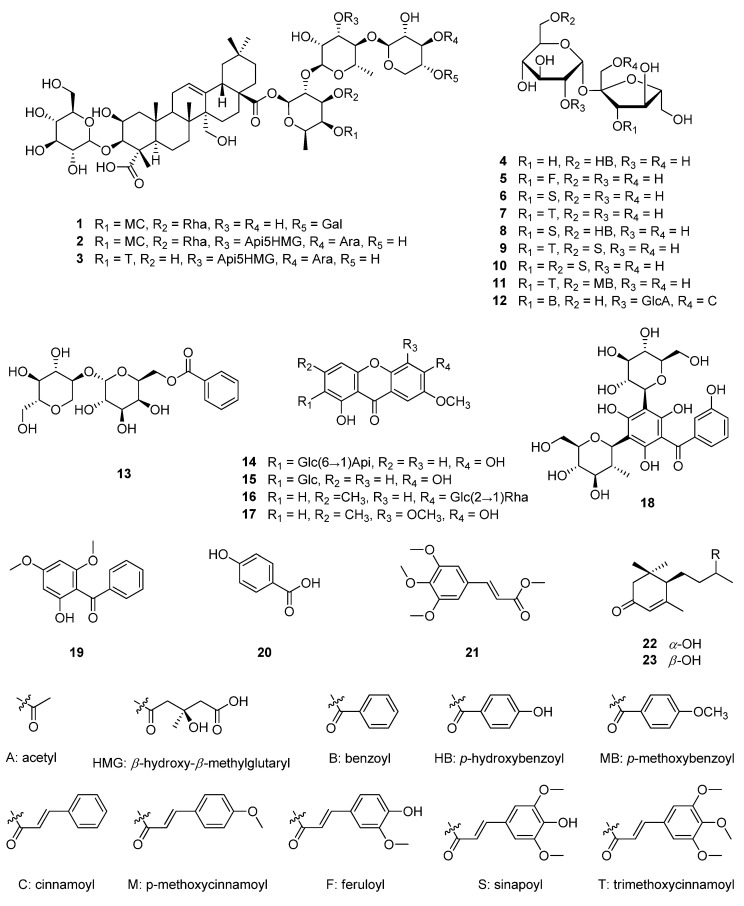
Compounds **1**–**23** isolated from the roots of *P*. *tenuifolia.*

In this study, twenty-three compounds were isolated from the roots of *P*. *tenuifolia*, including three saponins (**1**–**3**), ten phenylpropanoid sucrose esters (**4**–**12**), one benzoic acid sugar ester derivative (**13**), four xanthones (**14**–**17**), two hydroxy benzophenone derivatives (**18** and **19**), two phenolic derivatives (**20** and **21**), and two ionones (**22** and **23**) (Figure 1).

By comparing 1D-NMR (^1^H and ^13^C) spectra of the isolated compounds with previously reported data, the chemical structures were identified as onjisaponin B (**1**) [16], onjisaponin J (**2**) [17], onjisaponin Fg (**3**) [18], sibiricose A_3_ (**4**), sibiricose A_5_ (**5**), sibiricose A_6_ (**6**) [19], tenuifoliside A (**7**), tenuifoliside B (**8**), tenuifoliside C (**9**) [20], 3,6′-disinapoyl sucrose ester (**10**) [21], 3-*O*-(3,4,5-trimethoxy-cinnamoyl),6′-*O*-(*p*-methoxybenzoyl) sucrose ester (**11**), 1-*O*-(cinnamoyl),3-*O*-(benzoyl),2′-*O*-(6-*O*-acetyl-*β*-d-glucopyranosyl) sucrose ester (**12**) [22], polygalatenoside A (**13**) [14], polygalaxanthone III (**14**) [19], polygalaxanthone IV (**15**) [23], 7-*O*-methylmangiferin (**16**) [24], 3,4,5-trimethoxyxanthone (**17**) [25], tenuiphenone B (**18**) [26], hydrocotoin (**19**) [27], *p*-hydroxybenzoic acid (**20**) [28], 3,4,5-trimethoxycinnamic acid methyl ester (**21**) [29], blumenol C (**22**), and 9-*epi*-blumenol C (**23**) [30].

### 2.2. Effects of Compounds ***1**–**23*** on NO and PGE_2_ Production in RAW 264.7 Macrophages

**Figure 2:**
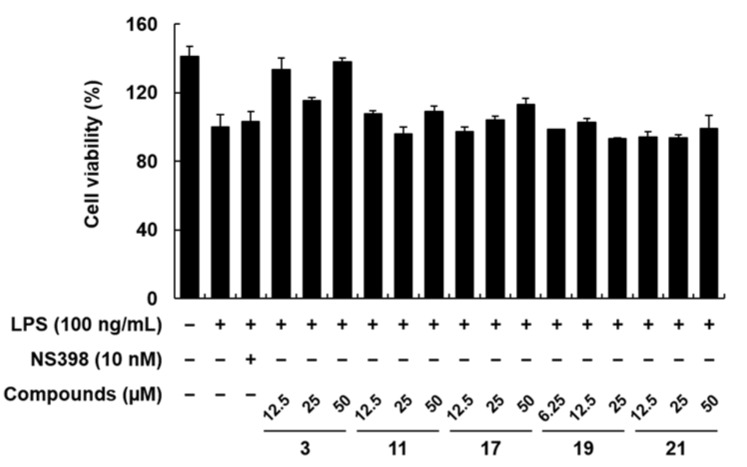
Effects of compounds **3**, **7**, **11**, **17**, **19**, and **21** on cell viability in LPS-stimulated RAW 264.7 macrophages. The cells were treated with compounds at concentrations of 6.25, 12.5, 25, and 50 μM and incubated for 24 h.

**Figure 3:**
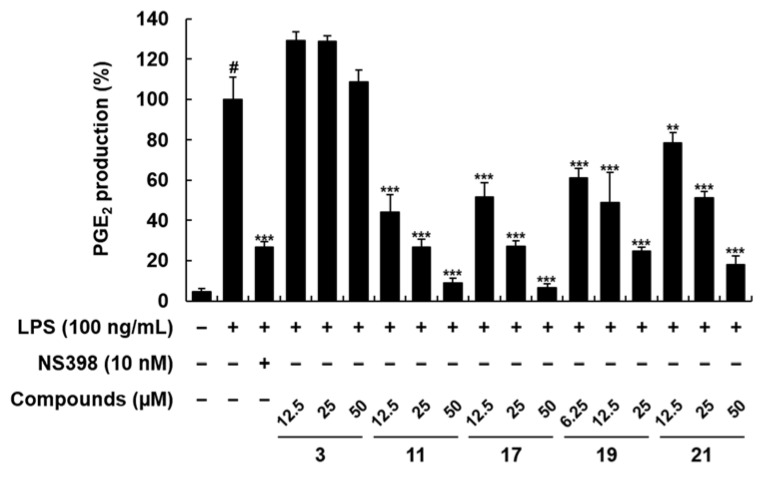
Effects of compounds **3**, **7**, **11**, **17**, **19**, and **21** on PGE_2_ production in LPS-stimulated RAW 264.7 macrophages. The cells were treated with compounds at concentrations of 6.25, 12.5, 25, and 50 μM and incubated for 24 h. NS398 was used as a positive control. Values are expressed as means ± SD. # *p* < 0.05 vs. non-LPS-induced control group; ** *p* < 0.01, and *** *p* < 0.001 as compared to the LPS-only-induced group.

The compounds **1**–**23** obtained from the roots of *P*. *tenuifolia* were evaluated for their inhibitory effects of LPS-stimulated NO and PGE_2_ production in RAW 264.7 macrophages (Table 1). Among these compounds, onjisaponins B (**1**) and J (**2**) were not subjected to further experiments since the cell viability of onjisaponins B and J (**2**) was under 80%, indicating the toxicity of compounds (data not shown), whereas onjisaponin Fg (**3**), 3-*O*-(3,4,5-trimethoxy-cinnamoyl),6′-*O*-(*p*-methoxybenzoyl) sucrose ester (**11**), and 3,4,5-trimethoxycinnamic acid methyl ester (**21**) exhibited significant inhibitory effects on LPS-stimulated NO production with observed IC_50_ values of 24.62, 46.08, and 32.92 μM, respectively at non-toxic concentrations (Table 1; Figure 2). Meanwhile, 3-*O*-(3,4,5-trimethoxy-cinnamoyl),6′-*O*-(*p*-methoxybenzoyl) sucrose ester (**11**), 3,4,5-trimethoxyxanthone (**17**), hydrocotoin (**19**), and 3,4,5-trimethoxycinnamic acid methyl ester (**21**) presented strong inhibitory effects on PGE_2_ production, with observed IC_50_ values of 10.01, 13.56, 11.03, and 24.57 μM, respectively (Table 1; Figure 3). Of these active compounds, 3-*O*-(3,4,5-trimethoxy-cinnamoyl),6′-*O*-(*p*-methoxybenzoyl) sucrose ester (TCMB) (**11**) was chosen for detailed experiments related to inflammation since it showed the most potent inhibitory effect on PGE_2_ production, as well as significant inhibition on NO production.

### 2.3. Effects of TCMB on iNOS and COX-2 Protein Level in RAW 264.7 Macrophages

**Figure 4:**
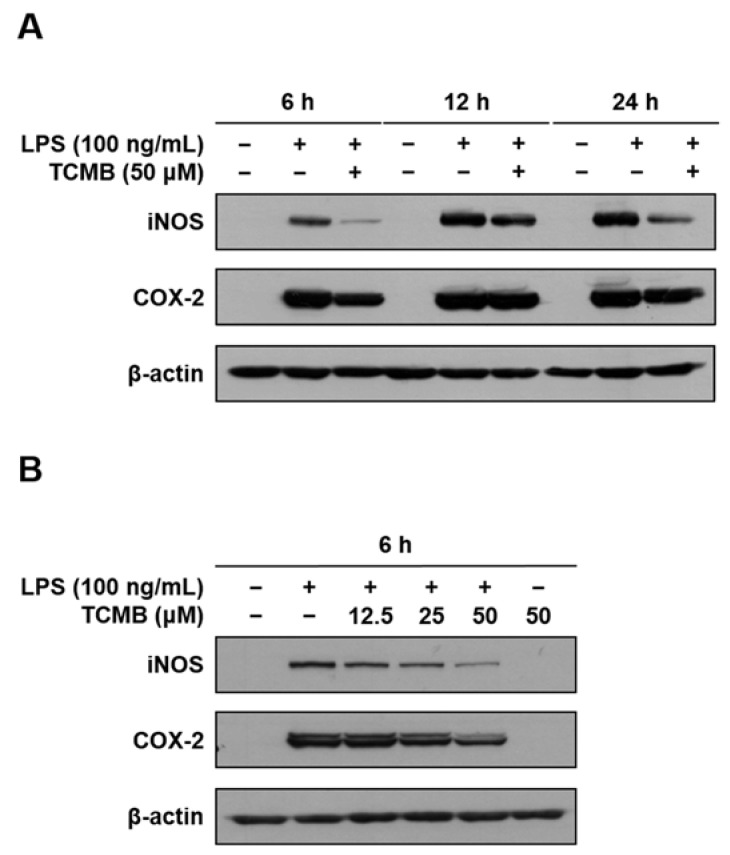
Effects of TCMB (**11**) on the protein level of iNOS and COX-2 in LPS-stimulated RAW 264.7 macrophages. The cells were pretreated with TCMB (**11**) at a concentration of 50 μM for the indicated time and then stimulated with LPS (100 ng/mL) (**A**). The cells were treated with TCMB (**11**) at concentrations of 12.5, 25, and 50 μM for 6 h and then stimulated with LPS (100 ng/mL) (**B**). The protein expression of iNOS and COX-2 was estimated by Western blotting using specific antibodies. β-actin was an internal control.

As TCMB (**11**) significantly inhibited the levels of NO and PGE_2_ production (Figure 2 and Figure 3), the protein levels of iNOS and COX-2 were estimated. After treatment with or without TCMB (**11**) and LPS for 6, 12, and 24 h, the protein expression of iNOS and COX-2 was upregulated by LPS treatment, whereas their expression was potently downregulated by the TCMB (**11**) treatment at a concentration of 50 μM (Figure 4A). Since TCMB (**11**) showed the strongest inhibitory effect against the protein expression levels of iNOS and COX-2 at 6 h, we further examined the ameliorating effect at various concentrations (12.5, 25, and 50 μM) of TCMB (**11**). As shown in Figure 4B, TCMB (**11**) decreased the protein levels of iNOS and COX-2 in a dose-dependent manner (Figure 4B). 

### 2.4. Effects of TCMB on iNOS and COX-2 mRNA Expression Levels in LPS-Stimulated RAW 264.7 Macrophages

**Figure 5:**
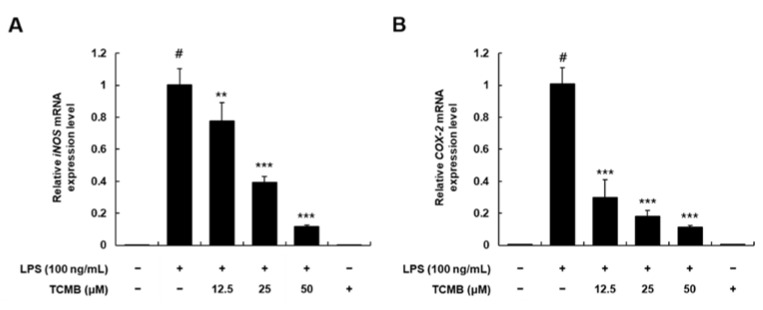
Effects of TCMB (**11**) on the mRNA expression levels of iNOS (**A**) and COX-2 (**B**) in LPS-stimulated RAW 264.7 macrophages. The cells were pretreated with compounds at concentrations of 12.5, 25, and 50 μM for 6 h and then stimulated with LPS (100 ng/mL). Values are expressed as means ± SD. # *p* < 0.05 vs. non-LPS-induced control group; ** *p* < 0.01 and *** *p* < 0.001 as compared to the LPS only-induced group.

To examine whether TCMB (**11**) affects the mRNA expression level of iNOS and COX-2, qRT-PCR was performed. The relative iNOS and COX-2 mRNA expression levels were elevated by LPS treatment, while TCMB (**11**) treatment (12.5, 25, and 50 μM) significantly diminished the relative LPS-stimulated mRNA expression levels of iNOS and COX-2 in a dose-dependent manner (Figure 5A,B).

### 2.5. Effects of TCMB on TNF-α, IL-1β, and IL-6 mRNA Expression Levels in LPS-Stimulated RAW 264.7 Macrophages

**Figure 6:**
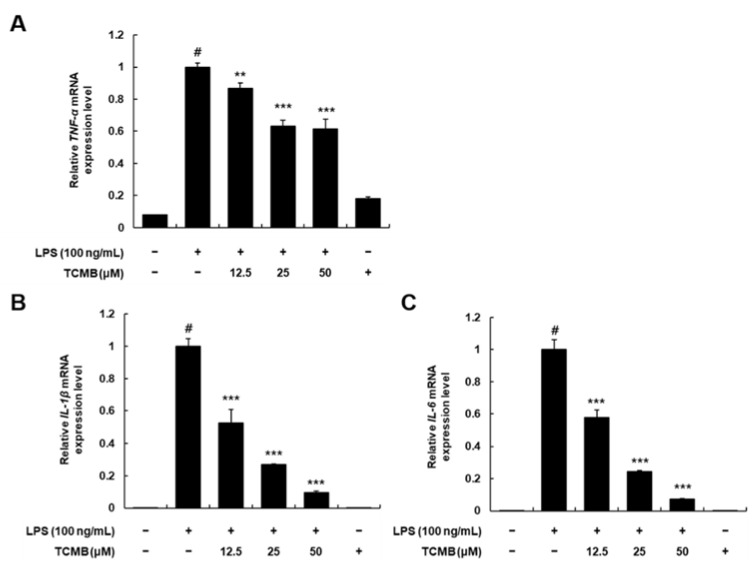
Effects of TCMB (**11**) on mRNA expression level of TNF-α (**A**), IL-1β (**B**), and IL-6 (**C**) in LPS-stimulated RAW 264.7 macrophages. The cells were pretreated with compounds at concentrations of 12.5, 25, and 50 μM for 6 h and then were stimulated with LPS (100 ng/mL). Values are expressed as means ± SD. # *p* < 0.05 vs. non-LPS-induced control group; ** *p* < 0.01 and *** *p* < 0.001 as compared to the LPS only-induced group.

Based on the data indicating the suppression effect of TCMB (**11**) on the production of proinflammatory mediators (NO and PGE_2_)_,_ we further estimated the relative mRNA expression levels of proinflammatory cytokines. As shown in Figure 6, we observed the dose-dependent suppression of TCMB (**11**) on the relative mRNA expression levels of TNF-α, IL-1β, and IL-6 in LPS-stimulated RAW 264.7 macrophages. These results suggest that further studies are needed to clarify the anti-inflammatory active site of TCMB (**11**). 

### 2.6. Molecular Docking with TCMB and iNOS/COX-2 Proteins

**Figure 7:**
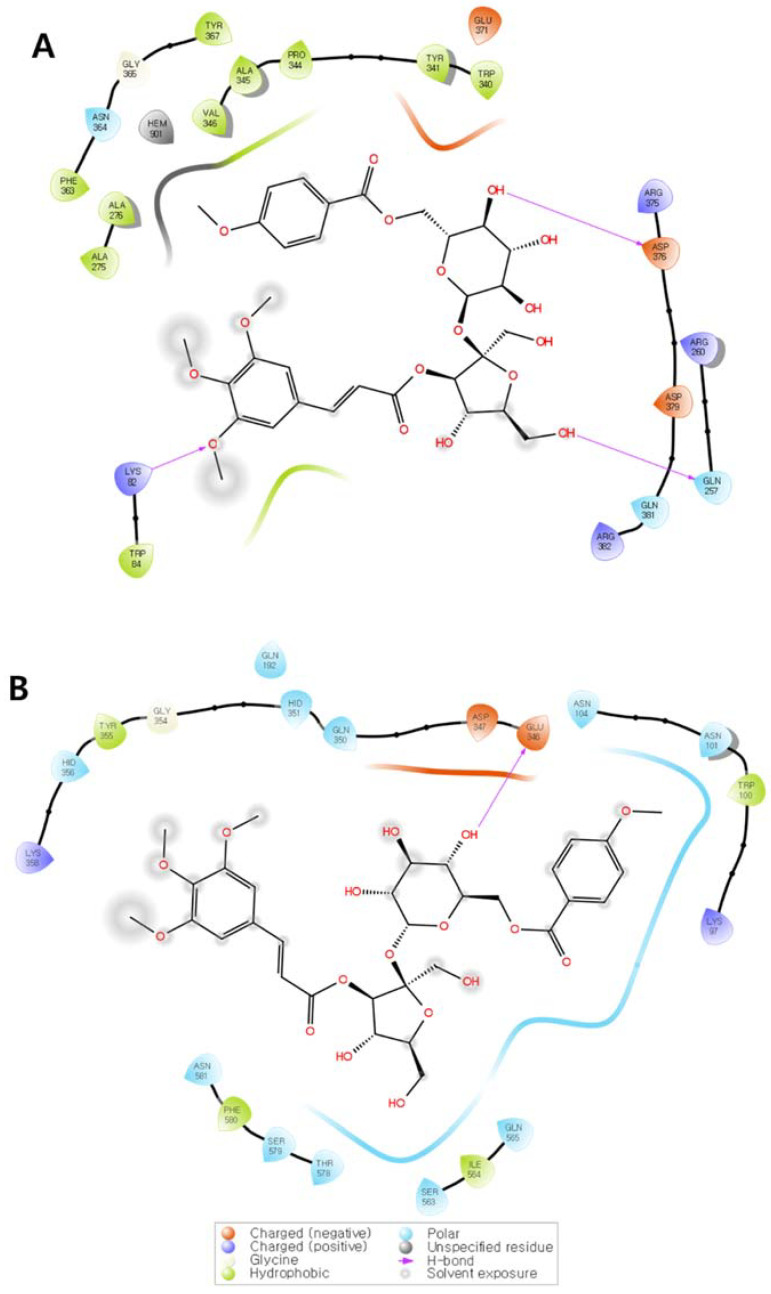
Receptor–ligand interaction diagram of TCMB (**11**) with the iNOS (**A**) and COX-2 (**B**) protein. The lowest docking position was selected for ligand interaction study (RMSD < 1.0). Only interacting residues were colored according to the following description: Negatively charged residue is red, positively charged residue is violet, hydrophobic residue is turquoise, polar residue is light blue, and glycine is white. HEM-901, a cofactor molecule, is colored by gray. Hydrogen bond interactions are indicated by pink arrows. Solvent exposure moiety is indicated by soft-edged gray circle. Visual illustrations of the receptor–ligand interaction diagrams were generated by Maestro software.

To further investigate how TCMB (**11**) interacts with the iNOS and COX-2 proteins, a molecular docking simulation was conducted in the active sites of each protein. Co-crystallized ligands IA2 for iNOS and S58 for COX, and positive controls NG-Monomethyl-L-arginine (L-NMMA) for iNOS and *N*-(2-cyclohexyloxy-4-nitrophenyl)methane sulfonamide (NS398) for COX-2, were utilized for comparison of binding affinity and docking position (Figures S1 and S2; Tables S1 and S2). At the lowest energy conformation, TCMB (**11**) showed a binding affinity of −10.4 kcal/mol (RMSD < 1.0) with iNOS. TCMB (**11**) interacted with residues ASP-376, GLN-257, and LYS-82 through hydrogen bonding, as shown in Figure 7A. ASP-376 and GLN-257 accepted a hydrogen bond from the sugar moiety of TCMB (**11**), whereas LYS-82 donated a hydrogen bond to the trimethoxycinnamoyl moiety. A hydrophobic pocket was exhibited by the association of hydrophobic residues from TRP-340 to VAL-346, ALA-275 to ALA276, PHE-363, and TYR-367. 

The lowest affinity of TCMB (**11**) with COX-2 protein was calculated as −7.1 kcal/mol (RMSD < 1.0). GLU-346 accepts a hydrogen bond from TCMB (**11**) in this conformation and is also involved in negatively charged residue with ASP-347 (Figure 7B). Positively charged residues were also revealed, such as LYS-97 and LYS-358. The most observed interaction was the association of several polar residues, including ASN-101, ASN-104, GLN-192, GLN-350, HID-351, HID-356, GLN-565, SER-563, THR-578, SER-579, and ASN-581.

## 3. Discussion

Since the potency of natural products with anti-inflammatory properties has been demonstrated, natural products have been employed continuously in the discovery for anti-inflammatory drug candidates. In a recent study, the root of *P*. *tenuifolia*, which has been traditionally used as an expectorant, was found to inhibit TLR4 and Myd-88 expression [11]. Moreover, *P*. *tenuifolia* roots suppress NF-κB translocation by blockade of IkappaB-α (IκB-α) degradation in LPS-stimulated BV2 cells [11]. It has also been reported that *P*. *tenuifolia* contains phytochemicals with anti-inflammatory properties. Tenuigenin, a triterpenoid from *P*. *tenuifolia*, inhibits mitogen-activated protein kinases (MAPKs) and NF-κB signaling while inducing Nrf2/HO-1 signaling in macrophages [31]. Tenuifoliside A (**7**), a unique phenolic sugar ester, has an anti-inflammatory effect in LPS-stimulated macrophages by inhibiting the c-Jun N-terminal kinase (JNK) and NF-κB signaling pathways [31]. In order to search for other phytochemicals with anti-inflammatory effects from the roots of *P*. *tenuifolia*, three saponins (**1**–**3**), ten phenylpropanoid sucrose esters (**4**–**12**), one benzoic acid sugar ester derivative (**13**) four xanthones (**14**–**17**), two hydroxy benzophenone derivatives (**18** and **19**), two phenolic derivatives (**20** and **21**), and two ionones (**22** and **23**) were isolated through repeated chromatography. To the best of our knowledge, blumenol C (**22**) and 9-*epi*-blumenol C (**23**) were isolated for the first time from the roots of *P. tenuifolia* in this study.

Proinflammatory mediators or cytokines are released by activated macrophages, causing inflammation-mediated diseases, including septic shock and arthritis [5,32]. Here, we tested the inhibitory impact of proinflammatory mediators on all extracted compounds from *P*. *tenuifolia* roots in an attempt to identify new inhibitors of proinflammatory mediators. An oligosaccharide ester, 3-*O*-(3,4,5-trimethoxy-cinnamoyl),6′-*O*-(*p*-methoxybenzoyl) sucrose ester (TCMB) (**11**), showed the most significant inhibitory effect on PGE_2_ production, with an IC_50_ value of 10.01 μM, and this is the first study to reveal that it has anti-inflammatory properties. In this work, however, another oligosaccharide, tenuifoliside A (**7**), exhibited a lower inhibition rate than TCMB (**11**), despite an anti-inflammatory mechanism being known.

Molecular docking with COX-2 provided another explanation for the PGE_2_ inhibition activity of TCMB (**11**). It showed significant binding affinity (−7.1 kcal/mol, RMSD < 1.0). Besides this, molecular dynamics simulations or kinetic analysis will be required to determine the stability of TCMB (**11**) with iNOS binding in a further investigation. In our experiment, the inhibitory effect of TCMB (**11**) on NO production was very weak, whereas simulations of direct binding to iNOS, iNOS protein, and mRNA expression revealed a strong binding affinity.

In the present research, it was observed that the inhibition rate of compounds on PGE_2_ and NO production were compatible with one another. However, the inhibition rate of NO production tended to be lower than that of PGE_2_ inhibition. Despite this tendency, TCMB (**11**) had a remarkable inhibitory effect on the protein and mRNA expression of iNOS and COX-2 in a dose-dependent manner. In addition, TCMB (**11**) significantly inhibited cytokines’ mRNA expression, including TNF-α, IL-1β, and IL-6. Further mechanistic studies and animal experiments will be able to prove its potential as an anti-inflammatory drug.

## 4. Materials and Methods

### 4.1. Plant Material

The roots of *Polygala tenuifolia* Willd. (Polygalaceae) were obtained at Neimenggu, China, in August 2017. The origin of the herbal material was identified by prof. Dae Sik Jang, and a voucher specimen (D170828001) was deposited in the Herb Resource Bank of Traditional Korean Medicine (http://herb-bank.com; accessed on 16 November 2022), Kyung Hee University (Seoul, Republic of Korea).

### 4.2. Extraction and Isolation of Compounds from P. tenuifolia

The dried roots of *P. tenuifolia* (3.0 kg) were extracted three times with 70% MeOH at room temperature for 24 h, respectively. 

The 70% MeOH extract (1.0 kg) was chromatographed over Diaion HP-20 (10.0 × 59.0 cm) as stationary phase eluting with a MeOH:H_2_O gradient [0:1 (7.0 L), 4:6 (12.0 L), 6:4 (19.5 L), 7:3 (2.0 L), 8:2 (23.0 L), 9:1 (5.0L), 10:0 (7.0 L)] to afford 15 fractions (P1–15).

P4 was fractionated using Sephadex LH-20 column chromatography (CC; 6.5 × 49.0 cm) with MeOH:H_2_O mixture (6:4 *v/v*) and produced 16 fractions (P4-1~P4-16). P4-7 was further subjected to silica gel CC (230–400 mesh, 4.8 × 25.0 cm, EtOAc:acetone:H_2_O = 5:4:1 to 4:5:1 *v/v/v*) and generated 20 subfractions (P4-7-1~P4-7-20). P4-7-9 produced compounds **13** (50.4 mg) and **6** (259.0 mg) using silica gel CC (230–400 mesh, 4.8 × 25.0 cm, CH_2_Cl_2_:MeOH:H_2_O = 7:2.7:0.3 to 6:3.6:0.4 *v/v/v*). P4-12 produced 16 subfractions using silica gel CC (P4-12-1~P4-12-16; 230–400 mesh, 3.8 × 25.0 cm, EtOAc:acetone:H_2_O = 5.5:4:0.5 to 5:4:1 *v/v/v*). P4-12-4 was fractionated into 12 fractions (P4-12-4-1~P4-12-4-12) by medium-pressure liquid chromatography (MPLC) using Redi Sep-C18 cartridge (43 g, MeOH:H_2_O = 1.3:8.7 to 3:7 *v/v*) to obtain compounds **4** (29.9 mg), **5** (151.5 mg), and **7** (10.8 mg). Compound **18** (16.8 mg) was obtained from P4-12-9 by MPLC using Redi Sep-C18 cartridge (13 g, MeOH:H_2_O = 1:9 to 3.5:6.5 *v/v*). Compound **20** (17.9 mg) was purified from P4-16 using MPLC with Redi Sep-C18 cartridge (30 g, MeOH: H_2_O = 1:9 to 6.5:3.5 *v/v*), successively.

P5 was subjected to silica gel CC (230–400 mesh, 6.5 × 58.0 cm, MC:MeOH:H_2_O = 8:1.8:0.2 to 5:4.5:0.5 *v/v/v*) to produce 13 fractions (P5-1~P5-13). Compound **14** (1.04 g) was purified by recrystallization with MeOH from P5-10. P5-2 was fractionated into P5-2-1~P5-2-9 by MPLC using Redi Sep-C18 cartridge (130 g, MeOH:H_2_O = 3.5:6.5 to 5:5 *v/v*). Compound **9** (30.3 mg) was purified from P5-2-6 using MPLC (Redi Sep-silica 24 g, CH_2_Cl_2_:MeOH = 9.5:0.5 to 7.5:2.5 *v/v*). P5-3 was fractionated into P5-3-1~P5-3-16 by GLAS RP-18 CC (ODS-A 12 nm S-150 μm, 5.2 × 50.0 cm, MeOH:H_2_O = 3.5:6.5 to 6:4 *v/v*). Compound **8** (352.5 mg) was purified from P5-3-8 using MPLC with Redi Sep-silica (40 g, CH_2_Cl_2_:MeOH = 9:1 to 7:3 *v/v*). P5-4 was fractionated using Sephadex LH-20 CC (3.8 × 60.0 cm) with MeOH:H_2_O mixture (7:3 *v/v*) and produced 14 fractions (P5-4-1~P5-4-14). Compound **16** (9.4 mg) was purified by recrystallization with MeOH from P5-4-11.

P9 was fractionated using Sephadex LH-20 (5.8 × 50.0 cm) with MeOH:H_2_O mixture (7:3 *v/v*) and produced 11 fractions (P9-1~P9-11). P9-6 was further fractionated using silica gel CC (70–230 mesh, 4.5 × 45.0 cm, EtOAc:acetone:H_2_O = 8:1.5:0.5 to 5:4.5:0.5 *v/v/v*) and produced 18 subfractions (P9-6-1~P9-6-18). Compounds **9** (100.0 mg) and **12** (92.4 mg) were obtained from P9-6-9 and P9-6-14, respectively. Compounds **22** (3.3 mg) and **23** (3.5 mg) were generated from P9-6-2 by performing prep HPLC with Gemini NX-C18 110A column (250 × 21.2 mm i.d., 5 μm, Phenomenex). Compound **11** (48.0 mg) was isolated from P9-6-6 using MPLC with Redi Sep-C18 cartridge (30g, MeOH: H2O = 2:8 to 8:2 *v/v*). P9-9 was chromatographed over silica gel (230–400 mesh, 3.6 × 32.0 cm, EtOAc:acetone:H_2_O = 9:0.5:0.5 to 6:3.5:0.5 *v/v/v*) to afford 17 pooled fractions (P9-9-1~P9-9-17). Compound **15** (112.3 mg) was obtained by recrystallization with MeOH from P9-9-15.

P10 was fractionated using Sephadex LH-20 CC (5.8 × 56.0 cm) with MeOH:H_2_O mixture (7:3 *v/v*) and produced 12 fractions (P10-1~P10-12). P10-9 was further fractionated using silica gel CC (230–400 mesh, 3.8× 32.0 cm, *n*-hexane: EtOAc:MeOH = 4.5:5:0.5 to 2.5:7:0.5 *v/v/v*), producing compound **10** (34.3 mg).

P13 was fractionated using silica gel CC (230–400 mesh, 4.5 × 42.0 cm, CH_2_Cl_2_:EtOH:H_2_O = 5:4.5:0.5 *v/v/v*) and produced 10 subfractions (P13-1~P13-10). P13-5 was further fractionated into P13-5-1~P13-5-16 by GLAS CC (ODS-A 12 nm S-150 μm, 5.2 × 50.0 cm, MeOH:H_2_O = 4:6 to 9:1 *v/v*) to give yielding compounds **1** (75.8 mg), **2** (158.0 mg), and **3** (25.8 mg).

P15 was separated by Sephadex LH-20 CC (5.8 × 56.0 cm) with MeOH:H_2_O mixture (8.5:1.5 *v/v*) and gave 16 fractions (P15-1~P15-16). Fraction P15-7 was fractionated into P15-7-1~P15-7-7 by MPLC (Redi Sep-silica 80 g, *n*-hexane:EtOAc = 9.5:0.5 to 6:4 *v/v*) to purify compounds **19** (9.7 mg) and **21** (15.5 mg). Fraction P15-13 produced compound **17** (8.3 mg) after using MPLC (Redi Sep-C18 26 g, MeOH:H_2_O, 7:3 to 8.5:1.5 *v/v*).

### 4.3. Cell Culture and Cell Viability Assay

The RAW 264.7 macrophage cells were obtained from the Korea Cell Line Bank (Seoul, Republic of Korea). These cells were cultured with Dulbecco’s modified Eagle’s Medium (DMEM) containing 10% fetal bovine serum and 1% penicillin and seeded in 24-well plates at a density of 2 × 10^5^/mL. After 24 h of incubation, cells were incubated with compounds at concentrations of 6.25, 12.5, 25, and 50 μM and then stimulated with LPS 100 ng/mL. After incubation for 24 h, the cell viability was estimated by MTT assay. A total of 20 μL of MTT solution (5 mg/mL) was added to each well and then incubated for 2 h. The supernatant was removed and remained formazan was dissolved with DMSO. The absorbance was determined by using microplate reader (Molecular Devices Inc., San Jose, CA, USA) at 540 nm.

### 4.4. Measurement of NO and PGE_2_ Production

The RAW 264.7 macrophage cells were seeded in 24-well plates at a density of 2 × 10^5^/mL. After incubation for 24 h, cells were pretreated with compounds at concentrations of 6.25, 12.5, 25, and 50 μM for 1 h and then stimulated with LPS 100 ng/mL for 24 h. The cultured media were collected to estimate NO and PGE_2_ production. To observe the NO production, 100 μL of cultured media was transferred to 96 wells, and then Griess reagent was added to each well. _L_-NIL (40 μM) was used as a positive control for the NO assay. The estimation of PGE_2_ production was performed using PGE_2_ enzyme-linked immunosorbent assay (ELISA) Kit (Enzo Life Sciences). NS398 (10 nM) was treated as a positive control for the PGE_2_ assay. The absorbance was determined by using microplate reader (Molecular Devices Inc., San Jose, CA, USA) at 504 nm and 405 nm for NO production and PGE_2_ production, respectively.

### 4.5. Western Blot Analysis

The RAW 264.7 macrophage cells were seeded in a culture dish at a density of 5 × 10^5^/mL. Cells were pretreated with 50 μM of TCMB (**11**) for 1 h and then stimulated with LPS 100 ng/mL for 6, 12, and 24 h. After incubation, the cultured media were removed and cells were washed with PBS. Whole protein was extracted by using PRO-PREP protein extraction solution (Intron Biotechnology, Seoul, Republic of Korea). Extracted proteins were quantified by Bio-Rad protein assay using a reagent (Bio-Rad Laboratories Inc., Hercules, CA, USA). The whole protein (25 μg) was separated by SDS-PAGE using 10% acrylamide gel and then transferred to the PVDF membrane. The blots were incubated with iNOS (sc-650, Santa Cruz Biotechnology, Santa Cruz, CA, USA) and COX-2 (No: 160106, Cayman Chemical, Ann Arbor, MI, USA) antibodies diluted in 5% skim milk with 1:1000 and 1:5000 ratio, respectively. After incubation at 4 °C for 18 h, the blots were washed 3 times using TBS/T. The washed blots were then incubated for 2 h with horseradish peroxidase-conjugated secondary antibody diluted in 5% skim milk with 1:2000 ratio and washed 3 times using TBS/T. The washed blots were developed using an ECL chemiluminescence substrate (Santa Cruz Biotechnology, Santa Cruz, CA, USA). The developed blots were then visualized with Amersham Hyperfilm ECL (GE Healthcare Life Sciences, Chicago, IL, USA).

### 4.6. Preparation of RNA and qRT-PCR

The RAW 264.7 macrophage cells were seeded in a culture dish at a density of 5 × 10^5^/mL. After incubation for 6 h, cells were pretreated with 12.5, 25, and 50 μM of TCMB (**11**) for 1 h and then stimulated with LPS 100 ng/mL for 6, 12, and 24 h. The cultured media were removed and cells were washed with PBS. Total mRNA was extracted by using Easy Blue^®^ kits (Intron Biotechnology, Seoul, Republic of Korea) and synthesized to cDNA using 0.5 mg/mL random oligonucleotide primers (Promega, Madison, WI, USA) and TOPscriptTM RTDryMIX (Enzynomics, Daejeon, Republic of Korea). PCR amplification was analyzed by using the incorporation of SYBR green (TaKaRa, Shiga, Japan) and primers for iNOS (forward strand 5′-AAC ATC CTG GAG GAA GTG GG-3′; reverse strand 5′-GCT GTG TGG TGG TCC ATG AT-3′), COX-2 (forward strand 5′-TGC TGT ACA AGC AGT GGC AA-3′; reverse strand 5′-GCA GCC ATT TCC TTC TCT CC-3′), TNF-α (forward strand 5′-AGC ACA GAA AGC ATG ATC CG-3′; reverse strand 5′-CTG ATG AGA GGG AGG CCA TT-3′), IL-1β (forward strand 5′-ACC TGC TGG TGT GTG ACG TT-3′; reverse strand 5′-TCG TTG CTT GGT TCT CCT TG-3′), and IL-6 (forward strand 5′-GGG ACT GAT GCT GGT GAC AA-3′; reverse strand 5′-CCA CGA TTT CCC AGA GAA CA-3′). All primers were purchased from Bioneer (Seoul, Republic of Korea).

### 4.7. Statistical Analysis

All results are expressed as the mean ± SD. Statistical significance was analyzed by using one-way analysis of variance (ANOVA) and Dunnett’s post hoc test using GraphPad Prism software (GraphPad Software, Inc., San Diego, CA, USA). # *p* < 0.05 vs. non-LPS-induced control group; * *p* < 0.05, ** *p* < 0.01, and *** *p* < 0.001 as compared to the LPS-only-induced group. *p*-values below 0.05 are considered significant.

### 4.8. Molecular Docking Study

Molecular docking studies were performed by a previously described method [33]. iNOS (PDB ID: 3E6T, resolution: 2.5 Å) and COX-2 (PDB ID:6COX, resolution: 2.8 Å) were downloaded from the RCSB PDB database. TCMB (**11**) was minimized with MMFF94 force field by Chem 3D pro 14.0. Proteins and ligands were prepared using AutoDock 4.2 software (The Scripps Research Institute, La Jolla, CA, USA). The active sites were determined by previously published data [34,35]. The molecular docking conformations were simulated with the hybrid Lamarckian Genetic Algorithm (LGA) by AutoDock vina software and receptor–ligand interaction diagrams were obtained by Maestro 12.9 software (Schrödinger, LLC, New York, NY, USA).

## 5. Conclusions

A total of 23 compounds were obtained from the roots of *P*. *tenuifolia* and were evaluated for their inhibitory effects of LPS-stimulated NO and PGE_2_ production in RAW 264.7 macrophages. Among these, 3-*O*-(3,4,5-trimethoxy-cinnamoyl),6′-*O*-(*p*-methoxybenzoyl) sucrose ester (TCMB) (**11**) exhibited significant inhibitory effects on both NO and PGE_2_ production. Western blot analysis revealed that TCMB (**11**) effectively downregulated the protein expression of iNOS and COX-2 in dose-dependent manner. Moreover, TCMB (**11**) showed inhibitory effect on the relative mRNA expression level of iNOS, COX-2, and cytokines (TNF-α, IL-1β, IL-6) significantly. Taken together, our data imply that TCMB (**11**) has an anti-inflammatory effect by inhibiting proinflammatory mediators. A molecular docking study showed that TCMB (**11)** has strong binding affinities with iNOS and COX-2 protein.

## Figures and Tables

**Table 1:** The inhibitory activities of compounds **1**–**23** obtained from the roots of *P. tenuifolia* on LPS-induced NO and PGE_2_ production in RAW 264.7 macrophages. _L_-NIL and NS398 are positive control for NO production and PGE_2_ production, respectively.

Compound	Inhibition Rate (%) ^a^ [IC_50_ (μM)]		Compound	Inhibition Rate (%) ^a^ [IC_50_ (μM)]	
	NO	PGE_2_		NO	PGE_2_
**1**	^b^ ND	^b^ ND	**13**	13.63	40.11
**2**	^b^ ND	^b^ ND	**14**	15.98	47.02
**3**	67.58 [24.62]	^c^ NE	**15**	13.92	44.50
**4**	^c^ NE	46.41	**16**	9.15	45.65
**5**	^c^ NE	50.54	**17**	47.50	94.44 [13.56]
**6**	^c^ NE	7.26	**18**	16.97	41.73
**7**	1.46	61.23	**19**	38.12	91.17 [11.03]
**8**	^c^ NE	36.25	**20**	9.63	37.50
**9**	25.45	65.05	**21**	56.53 [32.92]	79.39 [24.57]
**10**	18.56	45.52	**22**	9.61	46.68
**11**	68.21 [46.08]	93.39 [10.01]	**23**	8.89	38.67
**12**	11.86	45.40	**_L_** **-NIL**	57.87	^b^ ND
			**NS398**	^b^ ND	67.98

^a^ Cells were pretreated with **1**–**23** (50 μM) and LPS (100 ng/mL) for 1 h and incubated for 24 h. ^b^ ND: not detected. ^c^ NE: no effect.

## Data Availability

Not applicable.

## Supplementary Materials

The following supporting information can be downloaded at: https://www.mdpi.com/article/10.3390/plants11233307/s1, Figure S1. Docking poses of TCMB (**11**; pink carbon), a co-crystalized ligand (1A2; cyan carbon), and a positive control (NG-Monomethyl-L-arginine, L-NMMA; green carbon) with iNOS (PDB ID: 3E6T, RMSD < 1.0); Table S1. Binding affinity values of TCMB (**11**; pink carbon), a co-crystalized ligand (1A2; cyan carbon), and a positive control (NG-Monomethyl-L-arginine, L-NMMA; green carbon) with iNOS (PDB ID: 3E6T); Figure S2. Docking poses of TCMB (**11**; pink carbon), a co-crystalized ligand (S58; cyan carbon), and a positive control (N-(2-cyclohexyloxy-4-nitrophenyl)methane sulfonamide, NS398; green carbon) with COX-2 (PDB ID: 6COX, RMSD < 1.0); Table S2. Binding affinity values of TCMB (**11**; pink carbon), a co-crystalized ligand (1A2; cyan carbon), and a positive control (NG-Monomethyl-L-arginine, L-NMMA; green carbon) with COX-2 (PDB ID: 6COX, RMSD < 1.0).
