# Supplementary material for: Chemical Constituents of the Roots of Polygala tenuifolia and Their Anti-Inflammatory Effects
Source: Plants (Basel). 2022 Nov 30;11(23):3307. doi: 10.3390/plants11233307 (PMC9738712; doi:10.3390/plants11233307)
Supplement: Supplementary file 1 [file plants-11-03307-s001.zip › plants-2037279-supplementary.pdf]

Supplementary Materials

Figure S1. Docking poses of TCMB (**11**; pink carbon), co-crystalized ligand (1A2; cyan carbon), and a positive control (NG-Monomethyl-L-arginine, L-NMMA; green carbon) with iNOS (PDB ID: 3E6T, RMSD<1.0).

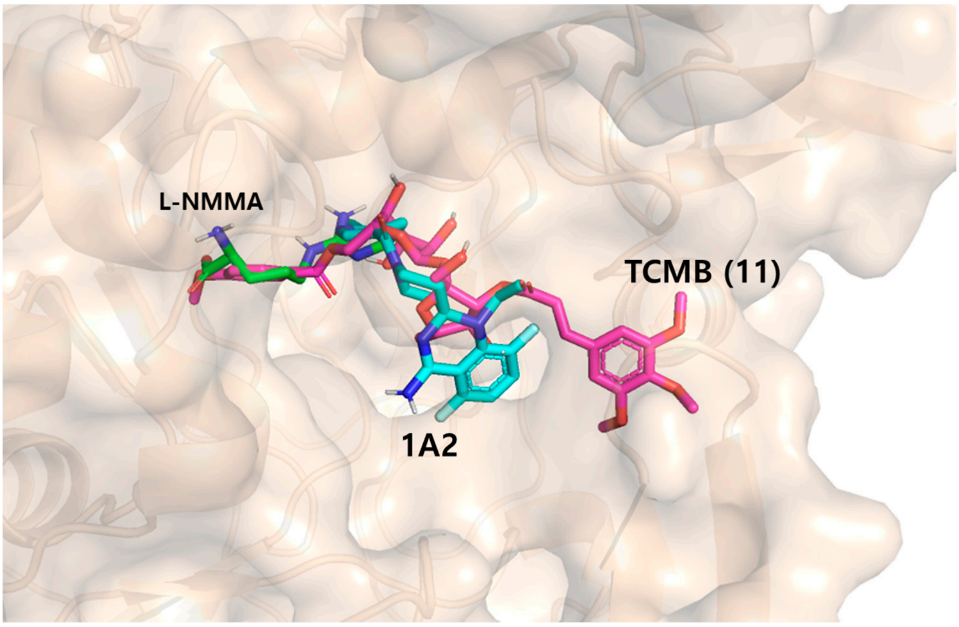

Table S1. Binding affinity values of TCMB (**11**; pink carbon), co-crystalized ligand (1A2; cyan carbon), and the positive control (NG-Monomethyl-L-arginine, L-NMMA; green carbon) with iNOS (PDB ID: 3E6T).

**Ligand**	**Binding affinity (kcal/mol)**
TCMB (**11**)	−10.4
1A2	−9.4
L-NMMA	−6.6

Figure S2. Docking poses of TCMB (**11**; pink carbon), a co-crystalized ligand (S58; cyan carbon), and a positive control (*N*-(2-cyclohexyloxy-4-nitrophenyl)methane sulfonamide, NS398; green carbon) with COX-2 (PDB ID: 6COX, RMSD<1.0).

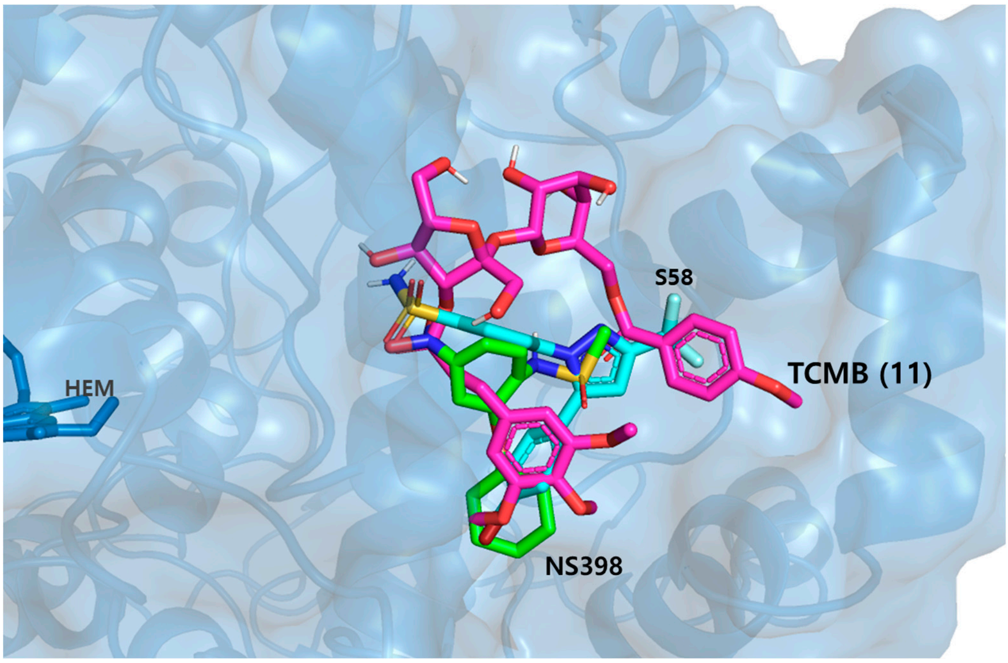

Table S2. Binding affinity values of TCMB (**11**; pink carbon), a co-crystalized ligand (1A2; cyan carbon), and a positive control (NG-Monomethyl-L-arginine, L-NMMA; green carbon) with COX-2 (PDB ID: 6COX, RMSD<1.0)

**Ligand**	**Binding affinity (kcal/mol)**
TCMB (**11**)	−7.1
S58	−7.3
NS398	−7.6
